# Research priority-setting for human, plant, and animal virology: an online experience for the Virology Institute of the Philippines

**DOI:** 10.1186/s12961-021-00723-z

**Published:** 2021-04-29

**Authors:** Reneepearl Kim Sales, Joseph Oraño, Rafael Deo Estanislao, Alfredo Jose Ballesteros, Ma. Ida Faye Gomez

**Affiliations:** Alliance for Improving Health Outcomes, Veria 1 Building, 62 West Avenue, West Triangle, 1104 Quezon City, Philippines

**Keywords:** Priority-setting, Agenda-setting, Virology, Health policy, Health planning, One Health

## Abstract

**Background:**

Viral pandemics have had catastrophic consequences on population health and economies. The Philippine government intends to establish the Virology Institute of the Philippines, one of the key areas of which will be virology research. This project aimed to develop the institute’s research agenda across the fields of human, plant, and animal virology.

**Methodology:**

Key considerations for the prioritization methodology were (1) the imminent establishment of the Virology Institute of the Philippines, (2) mobility restrictions caused by the coronavirus disease 2019 (COVID-19) pandemic, (3) the timeline to develop the research agenda, and (4) the need to separate the research agenda for the three fields of human, plant, and animal virology. The process was fully conducted online in four steps: stakeholder identification, soliciting research priorities, generating initial research priorities, and final prioritization consultations conducted on Zoom Pro.

**Results:**

Twenty-eight participants attended three online consultations between 21 and 27 July 2020 through Zoom Pro. Participants selected the research prioritization criteria and its weights, and used these to evaluate the research priorities. The final research agenda covers topics in epidemiology, diagnostics, surveillance, biosafety, and genomics.

**Conclusion:**

This initiative resulted in the first research agenda for the Virology Institute of the Philippines across the three fields of human, plant, and animal virology. An expert-driven process which places a premium on consensus-building facilitated through online platforms was the most feasible approach to develop the research agenda. This process resulted in an agenda aligned with the mandates of national research councils but leaves gaps on areas such as emerging infectious diseases. Pre-COVID-19 literature expressed apprehensions on the online medium that weakens social ties necessary for consensus. Our experience with changing the mode of consensus-building shows that users will continually adapt to technology. Online tools are currently able to address the limitations of the virtual space.

**Supplementary Information:**

The online version contains supplementary material available at 10.1186/s12961-021-00723-z.

## Background

The ongoing burden of viral diseases has led to the recognition of the increased importance of virology research. Viral pandemics have had catastrophic consequences on population health and economies. The World Bank estimates an economic loss of at least US$ 80 billion from six major outbreaks of highly fatal infections between 1997 and 2009 [1]. In 2020, coronavirus disease 2019 (COVID-19) escalated into a pandemic affecting 217 countries [2]. As of 1 February 2021, the Philippines has reported 527 272 cases, 10 807 deaths, and 487 574 recoveries from the virus [3]. The lockdown imposed to control its spread resulted in the highest unemployment rate and largest decline in the gross domestic product in the country’s history [4]. While several countries are beginning to ease lockdown measures to revive local economies, the threat to global population health remains until a vaccine is available [5].

Viral diseases of livestock also impact animal and agronomic health and food supplies [6]. In the Philippines, a trade loss of about 1 billion Philippine pesos (approximately US$ 21 million) was estimated after the country saw its first African swine fever outbreak in July 2019. This spread across major island regions, killing 251 450 pigs from culling and reduced national domestic production by 8.5% [7,8]. In July 2020, a case of the highly infectious H5N6 subtype of the influenza A virus was reported in the province of Pampanga, prompting a poultry farm to slaughter nearly 39 000 chickens to curb a bird flu outbreak [9]. In aquaculture, viral diseases also exact a heavy toll in production. In 2017, the tilapia lake virus killed 101 383 tilapia in Bulacan-based ponds alone [10]. In 2014, the white spot syndrome virus reduced the harvest of penaeid shrimps from 1 to 1.5 tons to as little as 200 kilos per hectare per fishpond [11]. The papaya ringspot virus reduced the yield of the small-scale papaya industry by 80% in the Southern Tagalog area, worth almost 60 million pesos in 1994 [12].

The intent of the Philippine government to establish the Virology Institute of the Philippines (VIP) was first made public through an announcement by the Secretary of the Department of Science and Technology (DOST) on 22 May 2020. In his statement, the VIP is “*envisioned to be the premier research institute in the field of virology encompassing all areas in viruses and viral diseases in humans, plants, and animals*” [13]. On 26 May 2020, Senate Bill No. 1543 was filed, seeking the establishment of the VIP. Citing the country’s current capacity to respond to the COVID-19 pandemic and viruses affecting livestock and plants, the VIP is expected to [14] (1) conduct molecular and biotechnology research, (2) develop diagnostics, vaccines, and therapeutics, (3) participate in international networks and databases of virus infections, and (4) operate a virus gene bank, genome laboratory, and reference laboratory.

The DOST has two offices that are involved in the setting up of the VIP—the Philippine Council for Health Research and Development (PCHRD) and the Philippine Council for Agriculture, Aquatic, and Natural Resources Research and Development (PCAARRD). These councils coordinate, evaluate, and monitor a network of institutions, including higher education institutions, that conduct research [15,16]. The PCHRD is focused on human health, while PCAARRD is open to all topics related to crops, livestock, and aquatic resources, and does not focus on animal or plant health, whether domesticated or wild [98]. The work of PCAARRD is on enhancing productivity and the management of resources, including for coconut, cacao, shrimps, and livestock.

Research priority-setting (RPS) is an essential part of managing a health system, and the resulting research agenda helps ensure effective use of resources for optimal health impact [17]. Institutional research priorities that address the entire field of virology are few and far between due to the vastness of the field of study. A research agenda similar to the one envisioned for VIP was developed by the WHO Thematic Reference Group on Environment, Agriculture and Infectious Diseases of Poverty (WHO-TRG4). Published in 2013, the Research Priorities for the Environment, Agriculture and Infectious Diseases of Poverty focused on changes in global environment and agricultural systems, and their role in the re-emergence of infectious disease [18]. These research priorities were intended for global adoption and were not associated with a specific research institution serving to provide guidance on priority research gaps and needs that should be addressed.

The VIP is set to be an attached agency of the DOST. As such, it should be included as one of the specific programs that the DOST has to establish research priorities for [19]. This project aimed to develop the VIP research agenda for human, plant, and animal virology, commissioned by DOST-PCHRD.

## Methodology

### Key considerations for the RPS method

The key decision points for designing the RPS method were the following:

- The VIP has not yet been formally established.
- The agenda needed to be developed over only a 3-month period.
- Mobility and assembly restrictions due to the COVID-19 pandemic.
- At the request of PCHRD and PCAARRD, the research agendas for human, plant, and animal virology were to be generated separately. This meant conducting three separate RPS processes.

**Fig. 1:**
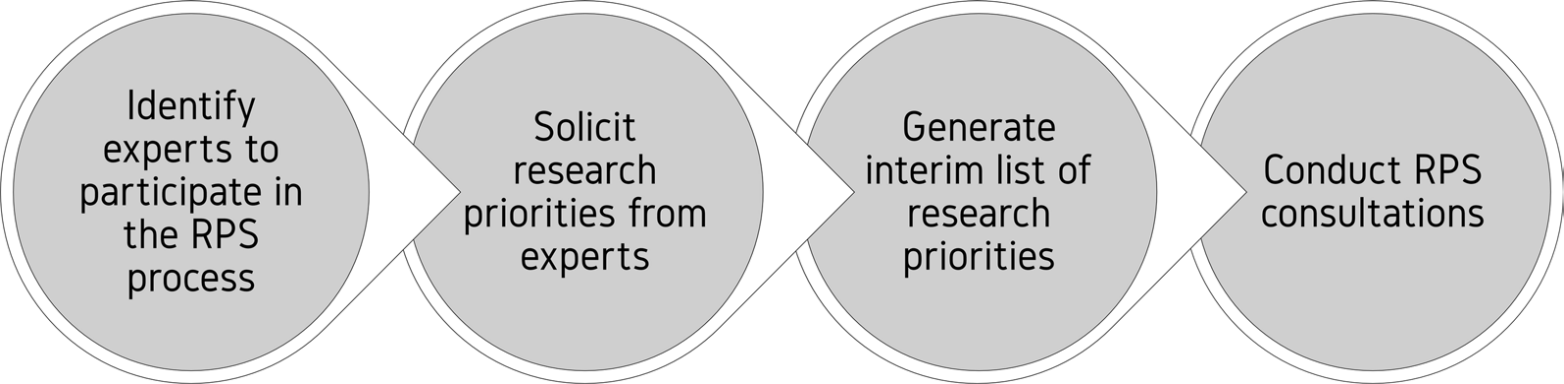
Research priority-setting process

Because of these realities, an expert-driven, transparent process conducted online was deemed most appropriate and feasible. The RPS was anchored on activities from the Philippine National Health Research System Guidelines for Health Research Prioritization, the James Lind Alliance framework, and the WHO Research Priorities for the Environment, Agriculture, and Infectious Diseases of Poverty (Fig. 1) [18,20,21].

### Stakeholder identification

Purposive and snowball sampling was used to ensure that stakeholders had relevant expertise in the fields of human, plant, or animal virology. An initial list of experts was provided by PCHRD. To expand this, experts were further identified through Google search and scoping of official websites of various government, industry/private corporations, research, and academic institutions across the country from 17 to 19 June 2020. Search terms used for identification of institutions and experts included “human, plant, and animal biochemistry”, “viral disease and pathology”, “agricultural biotechnology”, “plant breeding and genetics”, “aquaculture, fisheries, and ocean sciences”, “immunology”, “molecular medicine”, and “microbiology”. Contact with stakeholders was initiated via email. Additional stakeholders were snowballed as part of the online survey to solicit for research priorities. These recommendations were added to the stakeholder list and the additional stakeholders were invited to participate in the RPS process.

### Generating the initial list of research priorities

Research priorities structured as questions were solicited through two rounds of online surveys on Google Forms [22]. Informed consent to use their survey responses and participate in the study was obtained from the stakeholders in both surveys. The respondents were asked to provide personal and professional information, including their name, contact details, and institutional affiliation.

**Fig. 2:**
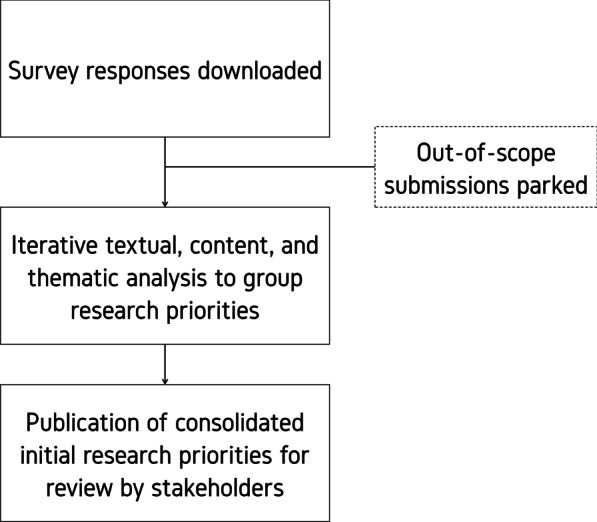
Process for generating the initial list of research priorities

Figure 2 shows how the initial list of research priorities was generated. The first survey was sent on 25 June 2020. Stakeholders were given 1 week to list a maximum of five research topics related to human, plant, or animal virology. Any virology-related research topic was eligible. After the first survey closed, each list of initial research priorities was reviewed by two individuals. The initial research priorities for human virology were reviewed by two males, one with a background in microbiology and the second with a background in medicine and RPS. The reviewers for the initial research priorities for plant and animal virology were two males, one with a background in public health and environmental health, followed by the second reviewer for the human virology initial research priorities. Disagreements were addressed through discussions with the principal investigator, a female with a background in laboratory science, international public health, and RPS.

Out-of-scope research priorities were research priorities that were deemed to be beyond the particular field of virology that is of interest in the survey, while ineligible research priorities consisted of research topics that were too broad. The researchers consolidated eligible research priorities, and indicative questions were formulated when appropriate. These indicative questions were considered the initial research priorities. In the second survey sent on 8 July 2020, stakeholders were encouraged to review the results of the first survey, which included the initial research priorities and submissions that were deemed ineligible. Stakeholders were given 1 week to further add five priorities to the initial research priority list.

### Preparing stakeholders for the RPS online consultations

Stakeholders were asked to confirm their attendance to the online consultations as part of the two online surveys. This early notice gave the stakeholders the opportunity to clear their schedules for participation in the consultations. A scheduled reminder was sent to stakeholders at least 7 days before the consultation.

To expedite discussions during the workshop, it was essential to minimize potential sources of confusion through grounding participants on similar premises and problem structures. Stakeholders who confirmed their participation received documents to prepare them for the consultation: (1) the initial list of research priorities and (2) a list of research prioritization criteria accompanied by clear definitions lifted from the 2013 WHO criteria for the Research Priorities for the Environment, Agriculture, and Infectious Diseases of Poverty, which would also serve as the choices of priority-setting criteria during the consultation [18]. From the initial list of priorities, participants were asked to select their two low-priority research priorities and two high-priority research priorities before attending the consultation.

### Pre-workshop preparations

Pre-workshop practice runs were conducted three times on the Zoom Pro meeting platform [23] to debug the slide deck, forms, and results computation, and to plan for contingencies during the small group and plenary discussions. For several activities during the workshop, participants were sent a link to surveys on Google Forms [22] that would generate data for Google Sheets [24]. Google Slides [25] were used, where inputs from the consultation would appear on the slide deck in close to real time. To cut down computation time of results, JavaScript [26] codes were prepared. Computation of interquartile ranges, standard deviation, measures of central tendency, and weighted averages were incorporated in the JavaScript codes.

### Research priority-setting consultation

The RPS consultations were rooted in the principle of consensus-building. The consultation design featured methods such as iterative round-robin discussions with allocated time for participants to express their views and individual anonymous voting. Consensus markers of a standard deviation of 1.5 or at least 70% agreement among participants were also applied [27]. The priority-setting was also rooted in the principles of multi-criteria decision analysis, which allows stakeholders to agree on assessment criteria, importance attached to each criterion, and how the information will be used to evaluate alternatives in priorities [28].

**Fig. 3:**
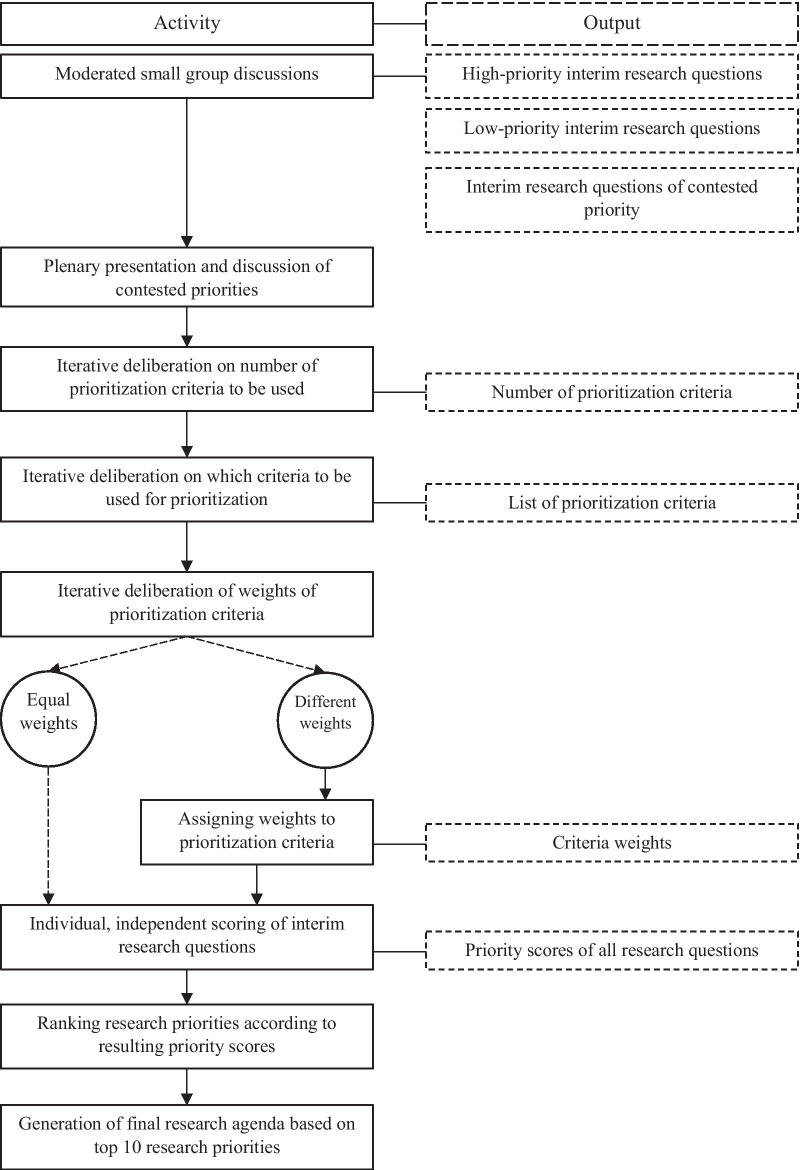
Summary of priority-setting workshop flow

The three online research priority-setting consultations were scheduled between 21 and 27 July 2020 via Zoom Pro. Each consultation was divided into two main phases: contextualization and prioritization. In contextualization, the progress of the research priority-setting process was presented, including its rationale, methodology, and the initial list of research priorities. Figure 3 summarizes prioritization, or the second phase of the workshop.

#### Moderated small group discussions

Participants were randomly assigned to breakout rooms in Zoom Pro. Following a round-robin format, participants presented two research questions each for highest and lowest priority based on the initial list of research priorities. After the small group discussions, intersections (research questions which appeared in both low- and high-priority lists) were raised for discussion. Each participant was given 1 minute to present their opinion on the intersections.

#### Priority-setting criteria

Selecting the priority-setting criteria was a three-step process. Each process was repeated up to three times if first round results did not meet the indicated consensus marker. First, participants had to determine the number of criteria to be used. Using the chat function in Zoom Pro, a Google Forms survey was sent for participants to complete. The median was selected if the results attained a standard deviation of 1.5 or less. After the number of criteria was set, participants then selected the prioritization criteria through Google Forms. For a criterion to be adopted, it had to receive at least 70% of the votes. If vacant slots remained, each participant had 1 minute to discuss their preferred criteria for inclusion. The survey was repeated until all criteria slots were filled. Finally, participants had to decide whether equal or different weights would apply to the criteria. This was determined through a Zoom Pro poll. The decision between equal or different weights was adopted if it received at least 70% of the votes. If the participants decided to apply different weights, a Google Forms survey was sent for participants to assign fractions of 100% to each criterion. The validity of the survey responses was monitored by ensuring that the total for each submission was equal to 100%. Following significant figure rules, the mean of the weights submitted by each participant was used.

#### Scoring the research priorities

The initial research priorities, criteria, and weights were compiled into a Google Forms survey. Participants were given 30 minutes to assess the research priorities on a scale of 1 to 10, with 1 as the lowest and 10 as the highest. Participants completed the survey individually, independently, and anonymously. The formula to compute the prioritization scores for both equal and unequal criteria weights is provided in Additional file 3. The top 10 research priorities according to their corresponding weighted average scores were presented to the participants as the final VIP research agenda.

## Results

A total of 124 stakeholders were invited to participate in the RPS process prior to the consultation (Table 1). Of these, 45 (36.29%) responded to the surveys.

**Table 1:** Stakeholder engagement summary

Research agenda	Initial number of stakeholders	Responded to survey 1	Snowballed stakeholders	Responded to survey 2	Total
Human virology	40	11	20	14 (10 new, 4 from initial sample)	21 out of 60
Plant virology	28	11	4	6 (1 new, 5 from initial sample)	12 out of 32
Animal virology	27	10	5	6 (2 new, 4 from initial sample)	12 out of 32

Twenty-eight stakeholders attended the three consultations (Table 2). Most of the consultation participants were female (61%), where they comprised the majority in human and plant consultations. While four groups of stakeholders were represented overall, these were not consistent across the three consultations. In each of the consultations, research and academe was the most highly represented, comprising 68% of the participants in total. Most participants were based in the Philippines (79%), but Filipino experts based in Australia, China, and the United States were also in attendance. Participants had 14 different areas of expertise in total, with virology the most highly represented expertise across the three consultations (25%).

**Table 2:** Characteristics of consultation participants

Characteristics	Total	Consultation		
		Human	Plant	Animal
Participants	28 (100%)	11 (39%)	8 (29%)	9 (32%)
Gender				
Female	17 (61%)	8 (47%)	6 (35%)	3 (18%)
Male	11 (29%)	3 (27%)	2 (18%)	6 (55%)
Group				
Government	4 (14%)	0 (0%)	2 (50%)	2 (50%)
Research and academe	19 (68%)	8 (42%)	5 (26%)	6 (21%)
Private corporations	4 (14%)	2 (50%)	1 (25%)	1 (25%)
Nonprofit	1 (4%)	1 (100%)	0 (0%)	0 (0%)
Location				
Philippines	22 (79%)	7 (32%)	7 (32%)	8 (36%)
Other (Australia, China, USA)	6 (21%)	4 (66%)	1 (76%)	1 (17%)
Area of expertise				
Aquatic pathology	1 (4%)	0 (0%)	0 (0%)	1 (100%)
Biodiversity	1 (4%)	0 (0%)	0 (0%)	1 (100%)
Biosafety and biosecurity	1 (4%)	0 (0%)	0 (0%)	1 (100%)
Biotechnology	1 (4%)	1 (100%)	0 (0%)	0 (0%)
Epidemiology	1 (4%)	1 (100%)	0 (0%)	0 (0%)
Immunology	3 (11%)	2 (67%)	0 (0%)	1 (33%)
Infectious disease	1 (4%)	1 (100%)	0 (0%)	0 (0%)
Livestock research	2 (7%)	0 (0%)	1 (50%)	1 (50%)
Microbiology	1 (4%)	0 (0%)	0 (0%)	1 (100%)
Molecular biology	3 (11%)	2 (67%)	0 (0%)	1 (33%)
Parasitology	1 (4%)	1 (100%)	0 (0%)	0 (0%)
Plant pathology	3 (11%)	0 (0%)	3 (100%)	0 (0%)
Vaccination research	2 (7%)	2 (100%)	0 (0%)	0 (0%)
Virology	7 (25%)	1 (14%)	4 (57%)	2 (29%)

Additional file 1 summarizes the generation of the initial research priorities. After review and consolidation, the two rounds of survey resulted in 75 initial research priorities: 27 for human, 24 for plant, and 24 for animal virology (see Additional file 2 for the complete initial list of research priorities).

The priority-setting criteria used in the three consultations is presented in Table 3. Both human and plant virology consultations opted for the same criteria and assigned equal weights. While five criteria were to be used for the animal virology consultation, two slots in the criteria selection process remained open after three rounds of voting. The participants opted to use six priority-setting criteria to accommodate both “Community focus” and “Potential for policy impact”.

**Table 3:** Priority-setting criteria selected in the three consultations and their weights

Criteria	Definition	Consultation and weights of criteria (in %)		
		Human virology	Plant virology	Animal virology
Interdisciplinarity	Involves three or more disciplines working continuously and interactively	20%	20%	18%
Impact on reduction of disease burden	Effectively targets diseases with high impact	20%	20%	26%
Innovation	Novel concept, methodology, and/or technology	20%	20%	19%
Feasibility/practicality	Achievable, credible, testable, replicable results	20%	20%	15%
Capacity-building potential	Improving knowledge and skill among service providers, policymakers, communities	20%	20%	N/A
Community focus	Research attends to, engages, empowers, and/or delivers benefits to communities involved	N/A	N/A	11%
Potential for policy impact	Policy relevance and proactive involvement of/influence on policy-makers	N/A	N/A	11%

Participants used the selected criteria and weights to evaluate and score the initial research priorities. This resulted in the first research agenda for the VIP across the three fields of human, plant, and animal virology, presented in Table 4. Epidemiology priorities were featured across the three research agenda. Medicines and diagnostics, such as vaccine production and assay development, were included in the human and plant research agenda. Monitoring and surveillance priorities for early warning systems or bioprospecting were featured in the human and animal priorities. Molecular and genome studies to better understand viruses were included in the plant and animal research agenda. Finally, biosafety and biodiversity research priorities were included for the animal research agenda.

**Table 4:** VIP research agenda for human, plant, and animal virology

Rank	Research agenda		
	Human virology	Plant virology	Animal virology
1	What mechanisms on collaborative data-sharing between the Virology Institute and other agencies or institutions are needed to allow for early warning system of impending outbreaks and to allow activating outbreak preparedness plans at all levels?	Detection, diagnosis, diversity, and molecular characterization of emerging and re-emerging plant diseases caused by viruses and viroids	What is the approach to surveillance and bioprospecting of viruses in all possible reservoirs, which have the potential to infect, or to evolve to infect, humans and/or be used for biological warfare or terrorism?
2	What are the circulating human viruses that have the most burden of disease, such as in terms of mortality rate, infectivity, or years lost to ill health or disability, to create a comprehensive molecular epidemiology (including collection, identification, and characterization through either cultures, genome sequencing, or biobanks) and knowledge of microbial pathogenesis of these relevant viral diseases in the Philippines?	What existing diagnostic tests or diagnostic tests under development, including those developed by local scientists, are available for application among food crops and other economically important plants, and how are they applied in subnational, regional, or local jurisdictions?	What is the current status of control and management of endemic viruses that affect poultry, livestock, and aquaculture, and what are better ways or strategies to mitigate the impact of these viruses?
3	What diagnostics can be developed to aid in the accurate and efficient diagnosis of viral infections, including the use of molecular diagnostics, and what mechanisms can be developed for monitoring field performance and quality assurance?	How should an integrated management of plant virus diseases be effected?	What are the epidemiologic, molecular, and virulence characteristics of all of the viral diseases that afflict aquaculture, including tilapia, shellfish, and crustaceans, and what are the ways to manage these diseases?
4	How can we develop multiple-pathogen diagnostic assay(s) for detection, diagnosis, and surveillance of disease with outbreak potential? (e.g., novel zoonotic emerging acute respiratory diseases/syndromes, and arthropod- and other vector-borne diseases)	What are effective control strategies for plant viruses?	What disease-causing viruses are present in wildlife species, especially priority species, and what is their molecular epidemiology?
5	How should the Institute establish or enhance medical countermeasures in the Philippines to include diagnostics, vaccines, and therapeutics against emerging viral infectious diseases?	Detection, diagnosis, and diversity of economically important plant viruses	In terms of biosafety, what is the appropriate approach for multi-species viruses, zoonotic viruses, and other viruses with dual use or potential risk for biological warfare or terrorism?
6	How should viral (biologically active and inactive) and viral antibody (IgG and IgM) diagnostics technology that can be applied at subnational (or regional) laboratories and/or points of use be developed?	What is the current status of control and management of endemic plant viruses in the country?	What are the potential hotspots and transmission factors for zoonoses?
7	How should surveillance and monitoring of novel and emerging pathogens and infectious diseases (including antigenic, genetic, environmental, and epidemiologic information) be programmed into the Institute?	What are the risks of virus and virus-like agents with introducing plant material and planting materials from crop production or importation?	What exotic viruses and virus-like agents are likely introduced with the importation of poultry, livestock, and aquaculture products?
8	What safe and efficient monitoring and surveillance strategies and methods can be developed for collecting, detecting/isolating high-consequence zoonotic viral pathogens in animals?	How can whole genome sequencing be used to better understand the characteristics, including the virulence, of viruses affecting economically important crops?	Should the Institute perform molecular characterization, including whole genome sequencing, of key poultry, livestock, and aquaculture viruses?
9	What are the important components in terms of necessary infrastructure, utilities, and security systems for a virology research institute to produce vaccines?	What point-of-care diagnostic tools and control strategies can be developed using serological- and molecular-based assays, including whole genome sequencing?	What are endemic and non-endemic poultry, livestock, and aquaculture viruses that seriously affect poultry, livestock, and aquaculture, and what is their prevalence and geographic distribution?
10	What new drugs from local sources can be developed against viral diseases, such as dengue, influenza, Japanese encephalitis, swine flu, COVID-19, HIV/AIDS, and other viral respiratory diseases?	What are novel conventional and molecular breeding methods applicable to crops for resistance to major plant virus diseases?	What are the molecular characteristics and ways to manage all viral diseases that afflict swine?

## Discussion

This exercise produced the first research agenda for the VIP, which is in the process of establishment in response to the COVID-19 pandemic and outbreaks in the Philippine agronomic sector. Forty-five stakeholders participated in the process of generating an initial list of research priorities and the final research agenda through consultations conducted online. The resulting research agenda produced priorities in the areas of epidemiology, diagnostics, surveillance, genomics, and biosafety.

### Online platforms for consensus-building for research priority-setting

Face-to-face meetings are the building blocks of consensus-based processes [29,30,31]. Real-time discussions in a shared physical space facilitate collaboration and allow appropriate adaptations to social, nonverbal, and feedback cues. It facilitates informal interactions that promote trust among participants [29,31,32]. However, opportunities to conduct group meetings were limited during the COVID-19 pandemic and necessitated the need to adapt the consensus-building process to online platforms.

The virtual conduct of consensus-building activities is not novel in the field of research priority-setting and does not prevent the advantages of physical meetings from being replicated [20,21]. Inputs were displayed on the slide deck to participants in almost real time to mimic the visual aids that traditionally complement consensus-building processes. Informal conversations were observed between participants during online breakout room sessions. Video could also supplement the lack of social cues in virtual spaces, although concerns about unreliable internet connections necessitated the restriction of video. The fear with the absence of social cues is a state of deindividuation, wherein participants express uninhibited and anti-normative behaviour, leaning towards greater conflict and hostility [32]. These were absent in our experience. Instead, we may have observed the positive effects of status and participation equalization by the same virtue of reduced inhibitions [30,32].

### Separation of human, plant, and animal virology research agendas

Virology is the study of viral diseases in humans, as well as in animals and plants that humans are concerned with, mainly livestock and crops [33]. The separation of human, animal, and plant viruses is useful, though there are perspectives such as One Health with a more ecologic framing [34,35]. One Health emphasizes that improving health and well-being requires a focus on crises that may originate in the interface between humans, animals, and the environments in which they interact [34]. Virus spillover in these interfaces leads to emerging infectious diseases [36,37,38,39,40,41,42].

The process of generating priorities surfaced several ways to organize the priorities gathered, including by viral genera, by potential viral host species, and around major activities such as diagnostics or vaccine development. Preemptively organizing the agenda around humans, plants, and animals reflects the framing that underlies existing institutions, namely the PCHRD and PCAARRD. This means that the interface of human populations with livestock, poultry, or wildlife is absent as a research priority.

A strict separation of research between plant, animal, and human viruses will leave crucial gaps in research on zoonoses and emerging infectious diseases, with serious implications on disease surveillance [43,44]. Moving forward, the VIP may benefit from building interdisciplinarity into its agenda and operations. This allows it to utilize combined expertise in human, animal, and potentially plant virology in conducting research on emerging infectious diseases and other crucial concerns.

### Stewardship of the VIP research agenda

To fulfil its mandate, Senate Bill 1543 stipulates that the VIP is duty-bound to implement national virology science policies and be the lead convener of the virology research agenda [14]. The development of the VIP research agenda was outsourced by DOST-PCHRD prior to the approval of the Bill and the formal establishment of the VIP.

Multiple RPS frameworks recognize leadership as an integral part of the RPS process and as a factor that influences its results [21,45,46]. Broadly, leadership has three major roles in RPS: (1) commit to the development, dissemination, and implementation of the research agenda, (2) sustain stakeholder participation by harnessing existing networks for engagement, and (3) have access to information and resources to support RPS until the post-implementation phase [21,46]. In outsourcing the development of the VIP research agenda, the methodology did not include plans for dissemination and implementation. Primarily, this was because the research agenda has no formally established owner and institution that can commit to these activities. While Senate Bill 1543 indicates the budget appropriations for the VIP, the Bill remains unapproved. This affected the finalization of the research agenda, where a list of available resources to the VIP would have eased feasibility assessment of the proposed research priorities.

### Scope of stakeholder involvement in the research priority-setting process

In determining the scope of stakeholder involvement in RPS, three questions have to be considered: (1) Who is the research agenda for?, (2) Whose perspectives do we want included?, and (3) Is the process intended to be accessible to all? [47]. The VIP is proposed to be the country’s premier virology research institute, and its research agenda can direct the efficient use of resources. In planning the RPS process, it was decided that only experts in human, plant, or animal virology would be invited to participate. This decision was made in part because of the short timeline to develop the research agenda, but also to secure early buy-in from researchers. Important steps in RPS were not undertaken under the premise that experts are best equipped with up-to-date knowledge and developments in virology, while also securing early engagement from individuals likely to lead or conduct research with the VIP. By comprising the majority of participating stakeholders, it is likely that the three VIP research agendas were products of inputs and perspectives from researchers. Once established, assuming resources are available, this allows the VIP to conduct research aligned with the current interests and capacities of virology researchers.

Most RPS frameworks recommend the involvement of a diverse set of stakeholders, cognizant of the fact that priorities may be overlooked due to the low or non-representation of vulnerable groups in particular [46,48,49,50,51]. Other stakeholder groups could have offered a different but relevant perspective on virology research priority areas based on their daily experiences [50]. As a result, the VIP research agenda may not be able to attain optimal impact of its research outputs by limiting the perspectives gathered during its development.

## Conclusion

Pre-COVID-19 literature expressed apprehensions of the online medium that weakens the social ties necessary for consensus. However, online tools are currently able to address the limitations of the virtual space. Our experience with changing the mode of consensus-building shows that users will continually adapt to technology. Additionally, the use of virtual spaces makes readily available the computational tools that facilitate the summarization and analysis of quantitative markers of consensus.

This initiative resulted in the first research agenda for the DOST-VIP across the three subfields of human, plant, and animal virology. The imminent establishment of the VIP, time frame provided to develop the research agenda, and restrictions caused by COVID-19 were key considerations in the RPS methodology. An expert-driven process which places a premium on consensus-building facilitated through online platforms was deemed the most feasible approach to develop the VIP research agenda. This process resulted in an agenda aligned with the mandates of PCHRD and PCAARRD but leaves gaps in areas such as emerging infectious diseases.

## Supplementary Information

**Additional file 1.** Summary of the generation of initial list of research priorities.

**Additional file 2.** Complete initial list of research priorities.

**Additional file 3.** Formula to compute research prioritization scores.

## Data Availability

The datasets generated and analysed for this study are available from the corresponding author on reasonable request.

## Abbreviations

DOST: Department of Science and Technology
PCAARRD: Philippine Council for Agriculture, Aquatic, and Natural Resources Research and Development
PCHRD: Philippine Council for Health Research and Development
RPS: Research priority-setting
VIP: Virology Institute of the Philippines
