# Supplementary material for: Research priority-setting for human, plant, and animal virology: an online experience for the Virology Institute of the Philippines
Source: Health Res Policy Syst. 2021 Apr 29;19:70. doi: 10.1186/s12961-021-00723-z (PMC8082216; doi:10.1186/s12961-021-00723-z)
Supplement: Supplementary file 1 — Additional file 1. Summary of the generation of initial list of research priorities. [file 12961_2021_723_MOESM1_ESM.docx]

|  | **First survey** | | | **Second survey** | | **Final consolidated priorities** |
| --- | --- | --- | --- | --- | --- | --- |
|  | Unique priorities | Out of scope or ineligible | Consolidated | Unique priorities | Out of scope or ineligible |  |
| Human virology | 47 | 4 | 17 | 36 | 11 | 27 |
| Plant virology | 38 | 8 | 17 | 14 | 1 | 24 |
| Animal virology | 36 | 8 | 18 | 20 | 1 | 24 |
