# Supplementary material for: Research priority-setting for human, plant, and animal virology: an online experience for the Virology Institute of the Philippines
Source: Health Res Policy Syst. 2021 Apr 29;19:70. doi: 10.1186/s12961-021-00723-z (PMC8082216; doi:10.1186/s12961-021-00723-z)
Supplement: Supplementary file 2 — Additional file 2. Complete initial list of research priorities. [file 12961_2021_723_MOESM2_ESM.docx]

| **No.** | **Initial research priorities for human virology** |
| --- | --- |
| 1 | What are the circulating human viruses that have the most burden of disease, such as in terms of mortality rate, infectivity or years lost to ill-health or disability, to create a comprehensive molecular epidemiology (including collection, identification and characterization through either cultures, genome sequencing or biobanks) and knowledge of microbial pathogenesis of these relevant viral diseases in the Philippines? |
| 2 | What are the important components in terms of necessary infrastructure, utilities and security systems for a virology research institute to produce vaccines? |
| 3 | How should surveillance and monitoring of novel and emerging pathogens and infectious diseases (including antigenic, genetic, environmental and epidemiologic information) be programmed into the Institute? |
| 4 | What effective antivirals can be produced through identification of other potential essential gene targets in viruses? |
| 5 | What in vitro and in vivo efficacy assay platforms can be established in the Philippines? |
| 6 | Can licensed vaccines be repurposed to target COVID-19? |
| 7 | How should the Institute establish or enhance medical countermeasures in the Philippines, to include diagnostics, vaccines and therapeutics, against emerging viral infectious diseases? |
| 8 | What new drugs from local sources can be developed against viral diseases, such as dengue, influenza, JE, swine flu, COVID-19, HIV/AIDS and other viral respiratory diseases? |
| 9 | Can the Institute be enabled to partner as contract research organizations (CROs) and contract manufacturing organizations (CMOs) with international pharmaceutical companies for investigational novel drug and vaccine development. |
| 10 | Should the Institute create a sequence database of mosquito arboviruses and other insect-specific viruses, to characterize the diversity of these viruses and to be used in early detection of circulating mosquito-borne viruses? |
| 11 | Can an effective vaccine against all 4 serotypes of dengue be developed? |
| 12 | Can a pan-coronavirus vaccine be developed using antigen discovery or engineering techniques? |
| 13 | What are yet undiscovered reservoirs of dormant or non-replicating HIV in human tissues? |
| 14 | What are vaccine prospects for hepatitis C? |
| 15 | What diagnostics can be developed to aid in the accurate and efficient diagnosis of viral infections, including the use of molecular diagnostics, and what mechanisms can be developed for monitoring field performance and quality assurance? |
| 16 | How should viral (biologically active and inactive) and viral antibody (IgG and IgM) diagnostics technology that can be applied at subnational (or regional) laboratories and/or points of use be developed? |
| 17 | What are current failures of direct-acting antivirals during clinical use? |
| 18 | What are other possible clinical and environmental control interventions for mosquito-borne arboviruses, and how should these control interventions be evaluated for their efficacy? |
| 19 | How can we systematically monitor the therapeutic efficacy (in vivo and in vitro) of first line and second line antiviral drugs for HIV and other viral diseases? |
| 20 | How can we develop multiple-pathogen diagnostic assay(s) for detection, diagnosis and surveillance of disease with outbreak potential? (e.g. novel zoonotic emerging acute respiratory diseases/syndromes, and arthropod- and other vector-borne diseases) |
| 21 | What is the infectious dose of SARS-CoV-2? |
| 22 | What safe and efficient monitoring and surveillance strategies and methods can be developed for collecting, detecting/isolating high-consequence zoonotic viral pathogens in animals? |
| 23 | What is the prevalence of children acquiring hepatitis B from vertical transmission despite being administered hepatitis B vaccine and hepatitis B immune globulin at birth? |
| 24 | What are the determinants of susceptibility and resistance to Hepatitis B and Hepatitis C infection among Filipinos (e.g. genetic predisposition) |
| 25 | What is the most effective early treatment for symptomatic and asymptomatic COVID-19 infection? |
| 26 | What is the seroprevalence of SARS-CoV-2 in areas with known low and high incidences of COVID-19 cases? |
| 27 | What mechanisms on collaborative data-sharing between the Virology Institute and other agencies or institutions are needed to allow for early warning system of impending outbreaks and to allow activating outbreak preparedness plans at all levels? |

| **No.** | **Initial research priority for plant virology** |
| --- | --- |
| 1 | Should the Institute create a comprehensive list of virus diseases of plants in the last 10 years, and how should it be compiled? |
| 2 | What existing diagnostic tests or diagnostic tests under development, including those developed by local scientists, are available for application among food crops and other economically important plants and how are they applied in sub-national, regional or local jurisdictions? |
| 3 | Detection, diagnosis, diversity, and molecular characterization of emerging and re-emerging plant diseases caused by viruses and viroids |
| 4 | What are novel conventional and molecular breeding methods applicable to crops for resistance to major plant virus diseases? |
| 5 | How can antibodies for application against major banana viruses be developed, produced and manufactured? |
| 6 | How should an integrated management of plant virus diseases be effected? |
| 7 | How can virus-free planting materials be produced, particularly for high value crops? |
| 8 | How can the reliability and efficiency of disease diagnosis based on disease records be increased? |
| 9 | What biocontrol and biocontrol application method using bacteriophages will be most effective against plant-pathogenic bacteria, such as soft-rot disease caused by *Pectobacterium* spp.? |
| 10 | What is the current status of control and management of endemic plant viruses in the country? |
| 11 | Detection, diagnosis and diversity of economically important plant viruses |
| 12 | What and where are the endemic plant viruses that seriously affect crops? |
| 13 | Is the cacao swollen-shoot virus present in the Philippines and what is its prevalence and mode of transmission? |
| 14 | What are the molecular bases of host-virus interactions that predict the effects of virus infection on plant growth and development? |
| 15 | What are the virulence determinants of carlavirus in rubber? |
| 16 | Using whole genome sequencing, what are the molecular characteristics of the banana bract mosaic virus and what are the virulence determinants and viral mechanisms against cellular responses to infection? |
| 17 | Should the Institute create a comprehensive database of published studies and unpublished studies on file which have been conducted for the past 10 years, and how should the database be compiled? |
| 18 | How can whole genome sequencing be used to better understand the characteristics, including the virulence, of viruses affecting economically important crops? |
| 19 | How can we enhance the capacity for virology research, particularly in terms of developing the information infrastructure and human resources necessary to generate the research? |
| 20 | What point-of-care diagnostic tools and control strategies can be developed using serological and molecular based assays, including whole genome sequencing? |
| 21 | What therapeutic products can be developed from plants that may be used to control plant viruses? |
| 22 | How might the effects of climate change affect the characteristics and distribution of plant viruses? |
| 23 | What are the risks of virus and virus-like agents with introducing plant material and planting materials from crop production or importation? |
| 24 | What are effective control strategies for plant viruses? |

| **No.** | **Initial research priorities for animal virology** |
| --- | --- |
| 1 | What are endemic and non-endemic poultry, livestock and aquaculture viruses that seriously affect poultry, livestock and aquaculture, and what is their prevalence and geographic distribution? |
| 2 | Should the Institute perform molecular characterization, including whole genome sequencing, of key poultry, livestock and aquaculture viruses? |
| 3 | Should the Institute create a comprehensive database of research studies on animal virus diseases in the past 10 years, to include those that have been published in national and international journals, and how should the database be compiled? |
| 4 | What is the extent of development of diagnostic technology, and how are these technologies applied, particularly at the point of use? |
| 5 | In terms of biosafety, what is the appropriate approach for multi-species viruses, zoonotic viruses and other viruses with dual use or potential risk for biological warfare or terrorism? |
| 6 | What is the approach to surveillance and bioprospecting of viruses in all possible reservoirs, which have the potential to infect, or to evolve to infect, humans and/or be used for biological warfare or terrorism? |
| 7 | What is the current status of control and management of endemic viruses that affect poultry, livestock and aquaculture, and what are better ways or strategies to mitigate the impact of these viruses? |
| 8 | What disease-causing viruses are present in wildlife species, especially priority species, and what is their molecular epidemiology? |
| 9 | What is the socio-economic impact of animal viruses affecting poultry, livestock and aquaculture? |
| 10 | What is the prevalence and distribution of decapod iridescent virus (DIV-1) in the country? |
| 11 | Should the Institute establish a facility capable of cryogenic electron microscopy? |
| 12 | What are the antigenic differences among African swine fever virus (ASFV) isolates circulating in the Philippines, and how can neutralizing antibodies be used for immunotherapy? |
| 13 | What are the molecular characteristics according to whole genome sequencing of avian influenza virus and what are the strains present locally? |
| 14 | Is the Ebola-Reston virus present in bat and swine populations in the Philippines? |
| 15 | What is the whole genome sequence of covert mortality nodavirus (CMNV) in shrimps and what are its virulence determinants? |
| 16 | What should the programmatic approach be to surveillance of animal viruses that affect poultry, livestock and aquaculture? |
| 17 | How is the progress in the field of animal virology in the country, considering major achievements and current status of institutions with virology research capabilities and mandates? |
| 18 | What have been the major constraints that inhibited commercialization, industrial production and development of animal viral vaccines in the country? |
| 19 | What exotic viruses and virus-like agents are likely introduced with the importation of poultry, pork, and aquaculture products? |
| 20 | Do arthropods play a role in the spread of virus and virus-like agents in livestock, poultry and aquaculture? |
| 21 | What are the potential hotspots and transmission factors for zoonoses? |
| 22 | What is the extent and effectivity of attenuated viruses, virus components, or probiotics in the control and management of viral diseases of livestock, poultry, and aquaculture? |
| 23 | What are the research institutions or groups in the Philippines that collect, catalogue, and monitor the culturing and storing of different animal viruses? |
| 24 | What are the characteristics and ways to manage all viral diseases that afflict swine? |
| 25 | What are the characteristics of and ways to manage all viral diseases that afflict aquaculture, including Tilapia, shellfish, and crustaceans? |
