# Supplementary material for: Research priority-setting for human, plant, and animal virology: an online experience for the Virology Institute of the Philippines
Source: Health Res Policy Syst. 2021 Apr 29;19:70. doi: 10.1186/s12961-021-00723-z (PMC8082216; doi:10.1186/s12961-021-00723-z)
Supplement: Supplementary file 3 — Additional file 3. Formula to compute research prioritization scores. [file 12961_2021_723_MOESM3_ESM.docx]

The prioritization score for each interim research priority was computed using the following formula when the participants decided to apply different weights:

$$PS_{i}=\frac{\sum_{j=1}^{m} \sum_{k=1}^{n} s_{ijk}w_{k}}{m}$$

where PS_i_: priority score of i^th^ interim research priority
 s_ijk_ : score of i^th^ interim research priority according to k^th^ criterion by j^th^ respondent
 w_k_ : weight of k^th^ criterion, expressed in decimal
 m  : total number of respondents
 n   : total number of criteria

When participants decided to apply equal weights to all criteria, an ordinary mean formula was applied:

$$PS_{i}=\frac{\sum_{j=1}^{m} \sum_{k=1}^{n} s_{ijk}}{m\cdot n}$$
